# Healthcare systems: what do we want?

**DOI:** 10.1590/S1516-31802011000400001

**Published:** 2011-05-05

**Affiliations:** I Associate Professor of the Discipline of Thoracic Surgery, Hospital das Clínicas, Faculdade de Medicina da Universidade de São Paulo.; II Trainee Physician in Tracheal Surgery and Therapeutic Respiratory Endoscopy in the Discipline of Thoracic Surgery, Hospital das Clínicas, Faculdade de Medicina da Universidade de São Paulo.

The United States does not have a public healthcare system with universal coverage. There are only some programs funded by the government, such as Medicare, for individuals over the age of 65 years, or Medicaid, for low-income individuals.^1-3^ Hence, most Americans need to obtain their own healthcare insurance plan, either through their employers or by funding healthcare themselves.^1,2^ The present healthcare system is criticized as being expensive and ineffective. In 2007, the United States spent US$ 2.2 trillion on medical care: equivalent to 16.2% of the gross domestic product (GDP).^1^ Over the same period, Spain spent 8% of its GDP on healthcare.^2^

The inadequacies of the American healthcare system were exposed in a study conducted in 2010,^4,5^ in which the present situation in 11 developed nations was compared. This study left the United States poorly placed in relation to others such as Australia, France and the United Kingdom. After consulting 19,700 adults in the United States, Germany, Australia, Canada, France, Netherlands, Norway, New Zealand, United Kingdom, Sweden and Switzerland, it was concluded that the American people were the ones who had the greatest need to survive without seeking a physician, because of the difficulty of paying for healthcare.

Because of the costs, adult Americans are much more inclined than those in the other ten industrialized nations to live without medical coverage. They have the greatest problems in dealing with the bills; they pay high prices even when they have medical insurance; and they get into disputes with their insurers regarding their coverage.^4,5^ The United States stands out among the other countries through having the worst healthcare experiences, considering that 33% of American adults recognize that they avoid seeking a physician when they are ill and also that they do not take the prescribed medications because of their price.^4,5^ In the Netherlands, this proportion is 5%, and in the United Kingdom, it is 6%.^4,5^ Moreover, 20% of Americans face "serious problems" in paying their medical bills, compared with 9% in France (the second country in this ranking), 4% in the Netherlands, 3% in Germany and 2% in the United Kingdom.^4,5^

The Canadian healthcare system, which is known as a single-payment model, differs from the American system mainly with regard to how it is funded.^6^ Instead of basing most of the system around private insurers, like in the United States, the Canadian government acts as a single source of payment. It collects money through taxes, negotiates with healthcare providers to reach agreements regarding expenditure, and then disburses the fees from a central public fund.^6^ This system could be better described as an interlinked set of ten provincial and three territorial health insurance plans.^7^ The system is known to Canadians as "Medicare" and it provides access to universal and comprehensive coverage of clinically necessary internal and external medical and hospital services.^6,7^

The role of the federal government in medical care involves setting and administrating national principles or norms for the medical care system (i.e. the Canada Health Act) and assisting in funding provincial medical care services, through fiscal transfers and carrying out functions that are constitutionally within its scope.^7^ The Canadian medical care system is focused on primary care physicians, who account for around 51% of all physicians active in Canada.^7^ These physicians generally form the initial contact with the conventional medical care system, and they control access to most specialists and similar professionals, hospital admissions, diagnostic analyses and prescription of medications.^7^

Canada does not have a socialized medical system with physicians employed by the government.^7^ Most physicians are private professionals who work in consultation offices, independently or in groups, and they enjoy a high level of autonomy. Private physicians are generally remunerated on the basis of payment for services provided, after presenting their fee invoices directly to the provincial insurance plan for payment.^7^

When Canadians need medical care, they usually go to the physician or clinic of their preference, where they present the healthcare insurance card that is issued to all residents of each province.^7^ Citizens do not pay directly for the insured services of hospitals and physicians, nor are they required to fill out forms for the insured services.^7^

The Canadian healthcare system has some significant advantages: the per capita healthcare expenditure, for example, is less than in the United States, and the coverage is universal.^6^ Life expectancy (79 years) is greater than in the United States. The disadvantages of the Canadian system include fewer physicians per 1,000 inhabitants than the average for the G7 (although not substantially less than in the United States), and less equipment like tomographs and magnetic resonance machines.^6^ There is also a slightly longer waiting time for undergoing certain high-complexity procedures.^6^

The Canadian and American systems differ greatly from the French system. In France, healthcare is one of the components of the social security system, which is based on compulsory contributions from employers and employees.^8^ Its organizational principles include: coexistence of the public sector alongside the private sector (operating either on a for-profit or a non-profit basis); free choice of professionals and healthcare establishments; autonomy to set up consultation offices; direct payment by users to the professionals and healthcare services, with partial reimbursement of expenditure; and freedom of prescription.^8^

In practice, the French system consists of a compulsory public insurance program that both remunerates private physicians for care provided and wields relative regulatory control over the cost of consultations and procedures.^8^ Because of the mixed nature of the system, it also makes available services of both public and private nature to users. The healthcare network consisted of approximately 3,000 hospital establishments in 2000, of which one third were public and two thirds were private. Among the private establishments, for-profit hospitals predominated.^8^

The fee table for healthcare professionals is negotiated between trade union entities and the social security system. It is based on actions implemented, which tends to increase the costs. In 2000, for example, a medical consultation conducted by a generalist cost 20 euros; by a cardiologist, 46 euros; and by a psychiatrist, 36 euros.^8^ Since the implementation of the "ticket modérateur" (copayment) system, which is an instrument aimed at controlling expenses and holding users partly responsible for the costs of the healthcare system, users have had to pay 30% of the cost of medical consultations, 40% of actions by auxiliaries, 40% of laboratory tests, 35% of expenditure on medical transportation and on glasses, along with a proportion of the cost of prescribed medications, ranging from 0 to 100%, according to the nature of the medication and the disease involved.^8^

The remuneration for medical activity in France is divided into three sectors. In sector 1, the fees are set as stipulated by the agreements with the social security system. In sector 2, physicians negotiate their fees with the social security system with an additional premium reflecting their specializations and titles; this occurs mainly in large cities.^8^ This additional premium is entirely for the user to pay. In 2000, 13.8% of the generalists and 37% of the specialists were following the norms for sector 2. Lastly, there is a small group of physicians in sector 3 who practice using freely set fees.^8^

State regulation within the healthcare sector covers different fields. For example, it covers control over and limitation of the number of professionals within certain specialties who are practicing in the country. This was done in France starting in 1971, through instituting a *numerus clausus*. At the time of its implementation, there were 8,588 students per year of medical courses, and this number fell to 4,000 by the start of the 21^st^ century.^8^

The Brazilian National Health System (Sistema Único de Saúde; SUS) was created through the federal constitution in 1988, to be a universal healthcare system for the population.^9^ However, the real SUS is still far from the constitutional SUS. In other words, there is an enormous distance between the dreams of the formulators of the Brazilian constitution and healthcare reform and the social practice of the public healthcare system.^10^ While the constitutional SUS proposed a universal public system for all Brazilians, expressed in terms of healthcare as everyone's right and the State's duty, the real SUS has become consolidated as a space destined for those who do not have access to healthcare insurance plans.^10^ Thus, a healthcare system in which three major subsystems coexist has been developing in Brazil: (1) SUS, the public subsystem, destined for 130 million Brazilians; (2) the private supplementary medical care system, destined for 40 million Brazilians who pay, by themselves or through employers, different healthcare insurance plan operators; and (3) the private direct disbursement subsystem, which both rich and poor Brazilians resort to, in order to purchase services by means of direct payment.^10^

The issue of public funding is the basis for the construction of the Brazilian social system.^10^ Data from the World Health Organization for the year 2001 show that Brazil spent little and badly on healthcare. The total per capita expenditure on healthcare in Brazil for that year was US$ 222.00, in contrast with US$ 603.00 in Uruguay and US$ 679.00 in Argentina, which attests to low healthcare expenditure in relation to other South American countries.^10^ Furthermore, the expenditure on the Brazilian healthcare system is of low quality, because of the proportion of public expenditure in relation to the total expenditure is only 41.6%. In countries that have constructed universal public systems, this proportion is greater than 70%.^10^

The issue of public healthcare funding depends on the kind of healthcare system that it is desired to construct: whether to invest in individualistic or self-interested values, as done by American society, which will lead to consolidation of SUS as a segment for the poor; or whether to invest in the values of social solidarity, which will turn SUS into a universal public system.^10^ One or other of these sets of values will form the SUS of the future. Brazilian society has still not made a definitive choice between them.^10^
